# Centering Communities of Color in the Modernization of a Public Health Survey System: Lessons from Oregon

**DOI:** 10.1089/heq.2023.0062

**Published:** 2023-09-20

**Authors:** Daniel F. López-Cevallos, Kusuma Madamala, Mira Mohsini, Andres Lopez, Roberta Suzette Hunte, Ryan Petteway, Tim Holbert

**Affiliations:** ^1^ Department of Health Promotion and Policy, School of Public Health and Health Sciences, University of Massachusetts Amherst, Amherst, Massachusetts, USA.; ^2^ Program Design and Evaluation Services, Oregon Health Authority, Public Health Division, Portland, Oregon, USA.; ^3^ Multnomah County Health Department, Portland, Oregon, USA.; ^4^ Coalition of Communities of Color, Portland, Oregon, USA.; ^5^ School of Social Work, Portland State University, Portland, Oregon, USA.; ^6^ Community Health Program, OHSU-PSU School of Public Health, Portland, Oregon, USA.

**Keywords:** communities of color, community leadership, health equity, public health modernization, surveillance

## Abstract

**Context:**

Public health survey systems are tools for informing public health programming and policy at the national, state, and local levels. Among the challenges states face with these kinds of surveys include concerns about the representativeness of communities of color and lack of community engagement in survey design, analysis, and interpretation of results or dissemination, which raises questions about their integrity and relevance.

**Approach:**

Using a data equity framework (rooted in antiracism and intersectionality), the purpose of this project was to describe a formative participatory assessment approach to address challenges in Oregon Behavioral Risk Factor Surveillance System (BRFSS) and Student Health Survey (SHS) data system by centering community partnership and leadership in (1) understanding and interpreting data; (2) identifying strengths, gaps, and limitations of data and methodologies; (3) facilitating community-led data collection on community-identified gaps in the data; and (4) developing recommendations.

**Results:**

Project team members' concerns, observations, and critiques are organized into six themes. Throughout this engagement process, community partners, including members of the project teams, shared a common concern: that these surveys reproduced the assumptions, norms, and methodologies of the dominant (White, individual centered) scientific approach and, in so doing, created further harm by excluding community knowledges and misrepresenting communities of color.

**Conclusions:**

Meaningful community leadership is needed for public health survey systems to provide more actionable pathways toward improving population health outcomes. A data equity approach means centering communities of color throughout survey cycles, which can strengthen the scientific integrity and relevance of these data to inform community health efforts.

## Context

Public health survey systems are important tools for informing public health programming and policy at the national, state, and local levels.^1–4^ For instance, the Behavioral Risk Factor Surveillance System **(**BRFSS), a collaborative project of the Centers for Disease Control and Prevention (CDC) and United States of America states and territories, is the largest, continuously conducted telephone health survey worldwide.^5,6^ In Oregon, a state located in the Pacific Northwest region of the United States, public health programming is partly guided by data gathered from the BRFSS (a state-based, random-digit-dialed telephone survey of the civilian, noninstitutionalized population 18 years of age and older) and school-based youth surveys (locally called the Student Health Survey—SHS, a school-based, anonymous, and voluntary health survey of 6th, 8^th^, and 11th graders conducted every 2 years).^7^

These large-scale survey systems help guide state and local decisions to target services, address emergent health issues, inform legislation, secure grant funding, and measure progress toward public health objectives. Federal funding for some programs depends on states using CDC's BRFSS survey. For example, the 2019 CDC notice of funding opportunity required Oregon's asthma program to use data from BRFSS Core survey.^8^ Each year, Oregon completes over 8000 BRFSS telephone surveys from a random sample of adults 18 years of age and older. For the past several years, most respondents (80%) participated using a cell phone.^7^

The Oregon Health Authority (OHA) relies heavily on the BRFSS and SHS for guiding policy and programmatic decisions. Over the past few years, awareness has grown about challenges of the BRFSS and SHS, particularly related to communities of color.^9^ For instance, CDC offers the possibility of adding an optional racial and ethnic oversample (at an additional cost) to the primary sample. Other challenges include the high cost to implement the oversample, lack of estimates for smaller geographic areas, lengthy survey completion times,^10^ concerns about representativeness and data validity/relevance for communities of color, and lack of community engagement in survey design, analysis, and interpretation of results or dissemination.^11^

In the case of BRFSS, several optional modules can be added (at a cost) to the core survey. For instance, the Reactions to Race (RTR) module (first piloted in 2002) consists of six questions assessing socially assigned race, race consciousness, and perceptions of and reactions to differential treatment by race/ethnicity in health care and work settings.^12,13^ Oregon included this module in its 2016 survey cycle. In the 2022 survey cycle, 27 states included the BRFSS RTR module, while 42 states included the newly developed Social Determinants and Health Equity module, which collects data on employment and economic stability, food and housing insecurity, transportation, and access to health care, among other topics.^14^

However, community feedback in Oregon about the RTR indicates that the module does not go far enough in capturing factors related to racialized experiences (e.g., hate crimes, stereotype threat) and does not address issues of economic insecurity, community violence, and institutional racism (e.g., involvement with the justice or child protective service systems).^11^

The coronavirus disease 2019 (COVID-19) pandemic has made evident several other challenges (such as disruptions on data collection and processing and variations on definitions of race/ethnicity across states), highlighting the urgency of data accuracy and consistency; timely data dissemination; minimizing burden and maximizing safety; and data relevance, particularly for communities of color.^15,16^ National discourse on transforming public health data systems is ongoing.^17,18^ To address some of these issues locally, OHA and community partners are using a data equity framework (rooted in antiracist and intersectionality principles) to guide public health modernization efforts.^19,20^

Public health modernization aims at creating an equity-centered public health system, including communicable disease protection, health promotion and disease/injury prevention, environmental health, and access to health services.^21^ The project team, consisting of OHA staff and community partners, used this opportunity to conduct a community-engaged process to elicit feedback on revising and updating Oregon's BRFSS and SHS systems. This collaborative journey demonstrates how data equity principles apply to survey modernization efforts. Project efforts were facilitated by OHA staff and the Research Justice Institute at the nonprofit Coalition of Communities of Color (CCC), whose work focuses on research and data justice and whose members include 19 culturally specific community-based organizations.^22^

## Approach

Using a formative participatory assessment approach, we describe the early stages of programmatic development and implementation of a system-wide survey modernization effort. Hence, one of our first decisions was to evaluate the design and implementation of the BRFSS and SHS, placing community engagement at the center of these efforts. The purpose of this community-centered project was to (1) better understand and interpret BRFSS and SHS data; (2) identify strengths, gaps, and limitations of BRFSS and SHS data and methodologies; (3) facilitate community-led data collection on identified gaps in the data and co-create more actionable survey questions; and (4) develop recommendations. In this article, we describe our work with African American, African Immigrant and Refugee, and Latinx populations as part of a broader effort to center communities of color in public health modernization efforts in Oregon.^9,21^

In the initial stages of this project, OHA staff assumed they could rely on existing community partnerships to find potential project team members. However, they realized that, while partnerships with community organizations existed within OHA units, they were effectively inaccessible and needed to be more cohesive and coordinated organization wide. Consequently, staff undertook outreach and recruitment efforts to find potential project team members. Project staff used a snowball methodology to develop a list of potential members for two culturally specific analytic project teams—one African American/African Immigrant and Refugee and one Latinx.

Between October 2019 and March 2020, project staff connected with community-based organizations that had previously collaborated with Multnomah County Health Department, the OHA Office of Equity and Inclusion, and other OHA offices (i.e., local and regional community-based organizations and leaders, including the CCC—our survey modernization project partner). They reviewed previous statewide internal and external community health data reports and a membership of the community-based Health Equity Researchers of Oregon group, to identify people with lived experience (African American, African Immigrant and Refugee, and Latinx) and experience in public health and/or research. Individuals who met these criteria were interviewed between October 2019 and March 2020. Nine individuals were invited to participate in two small (four to five person) culturally specific project teams (i.e., African American/African Immigrant and Refugee and Latinx). Contracts and compensation for each team member were established.

**FIG. 1.**
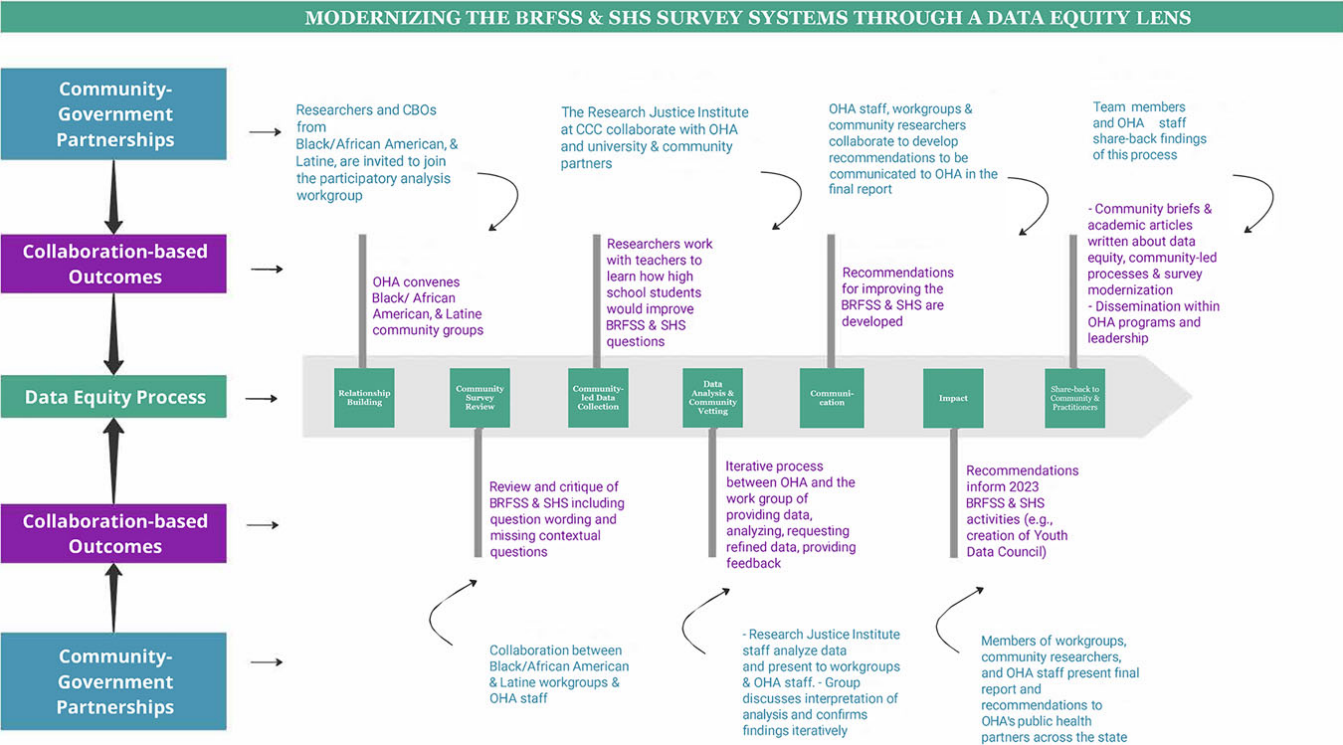
Snapshot of Oregon's data equity approach to public health survey modernization efforts with African American, African Immigrant and Refugee, and Latinx communities.

Teams met five times for 2 h each from May 2020 to April 2021 to review BRFSS and SHS data and methodologies, suggest and review additional requested analyses, discuss strengths, gaps, and limitations, identify topics for community-led data collection, review results of data collection, and develop recommendations (Fig. 1). Areas of concern developed organically through conversations with the project teams during group meetings.

The two project teams conducted the bulk of their work separately and came together for the last two meetings to review results of community-led data collection and provide recommendations for work in the future. The project teams decided to share their work together in one report because (1) the topic areas of interest/review overlapped significantly (e.g., mental/behavioral health and health care access) and (2) the COVID pandemic limited the capacity for community engagement, and there was a desire to limit/integrate requests of community groups.

During the scheduled meetings (May 2020–April 2021), project team members reviewed survey instruments, requested specific analyses of BRFSS and SHS data, interpreted results, identified knowledge gaps in the data, and suggested areas for community-led data collection. With guidance from the CCC facilitators, team members helped design community-led data collection, analysis, and interpretation of those results. Project teams explored (1) types of survey questions, (2) questionable wording, (3) whether data resonated with their experience and with local data, (4) concerns about the sample, (5) whether additional information was needed to understand the findings and provide important context that BRFSS and SHS lack, and (6) additional data sources relevant to the BRFSS and SHS data.

After reviewing the BRFSS and SHS data, project teams decided to focus the community-led data collection on two topic areas:

(1) Mental and behavioral health, especially access to care. These data were gathered through a statewide behavioral health survey of BIPOC communities.^23^
(2) Health of youth. Information about this issue came from youth at a Portland-area high school, who gave input into the design of SHS questions.

Throughout this process of engaging with community partners and the project teams, a common concern was that BRFSS and SHS reproduce assumptions, norms, and methodologies of a White dominant, individual-centered culture and in so doing, run the risk of misrepresenting racial and ethnic minoritized populations, ultimately further eroding trust, increasing harm, and re-traumatizing communities. We further address these concerns in the discussion section.

## Results

Project team members coalesced around six areas of concern: (1) Lack of meaningful context (centering individual responsibility without community/policy context; (2) missing an intersectional approach^19,20,24^; (3) need for actionable data (ability to drive policy and practice); (4) sample size/response rate (low number of respondents from communities of color); (5) integration of other data sources; and (6) translations and health literacy. These issues are bound together under one fundamental perspective: the BRFSS and SHSs are products of a dominant institutional culture that centers whiteness. As such, these data harm communities of color through misrepresentation and reinforcing narratives about individual shortcomings that perpetuate racialized stereotypes.^19,20^

First, team members expressed concern that survey questions do not collect meaningful contextual information and exclusively focus on individual behavior. These types of questions, presented without context, place the entire responsibility of outcomes on the individual. Team members desired questions that contextualized individual outcomes within underlying socioecological conditions, and that highlighted the role and responsibility of institutions—historically and currently—in creating, perpetuating, and exacerbating health inequities. Team members expressed that the current BRFSS and SHS instruments required additional questions to be able to provide enough context to interpret data findings correctly (see Table 1 for BRFSS examples and Table 2 for SHS examples).

**Table 1.** Behavioral Risk Factor Surveillance System Questions Versus Community-Led Questions About Health Care Access

BRFSS	Community-led data collection
Do you have any kind of health care coverage, including health insurance, pre-paid plans such as HMOs, government plans such as Medicare or Indian Health Services? Are you currently enrolled in the OHP, which is the State's Medicaid program? Do you have one person who you think of as your personal doctor or health care provider? Was the real time in the past 12 months when you needed to see a doctor, but could not because of the cost? About how long has it been since you last visited a doctor for a routine checkup?	Do any of the following prevent you or members of your family from seeking support from your CCO or other health provider with issues around stress, frustration, worry, anger, addiction, violence, and/or abuse? Please select all that apply. CCO/health provider is too far away Do not have access to transportation Do not have access to childcare Do not have consistent access to internet for virtual appointments Do not have health insurance Process for making an appointment with a provider is difficult Do not feel safe visiting my provider Provider cannot communicate in a language that I'm comfortable using Provider does not have the same cultural background as me The service(s) I/we need is not covered by my insurance The service(s) I/we need is not available near me Not aware of what services are available near me Information about services is not provided in a language that I am comfortable using Do not trust that my CCO/health provider will be respectful of my cultural values

BRFSS, Behavioral Risk Factor Surveillance System; CCO, Coordinated Care Organization; HMOs, Health Maintenance Organizations; OHP, Oregon Health Plan.

**Table 2.** Student Health Survey Questions Versus Community-Led Questions About School Absenteeism

SHS	Community-led data collection
During the past 12 months, how many days of school did you miss – for any reasons? because of physical health reasons? because of emotional or mental health reasons? How many days of school did you have unexcused absences (meaning you skipped or cut school)? Did you miss one or more hours of school due to any of the following reasons? I had a toothache or painful tooth; My mouth was hurting; I had to go to the dentist because of tooth or mouth pain; I had to go to the hospital emergency room because of tooth or mouth pain; I had a mouth injury from playing sports	What is causing you to miss school? (open ended) Do you have problems at home/outside of school? Are you doing ok? After each question add a “why” follow-up What is elevated above school? Why does it come up? Do you have other things to do other than school? What are things affecting you outside of school that keep you from being successful? In what ways does school feel unsafe to you? Is someone making fun of you or are there stressful conditions you want to avoid at school like students or teachers? What would make school a safer environment? What at schools feels welcoming/accepting? What does not?

SHS, Student Health Survey.

Second, it is essential to recognize that individuals exist within intersectional/overlapping structural conditions (e.g., gender, race/ethnicity, class, interpersonal and institutional racism) that shape people's everyday experiences and their ability to leverage power, resources, and opportunities.^20^ For instance, team members pointed out that inclusion of racial harassment in the SHS as simply a form of “bullying” is problematic because it minimizes the extent and depth of interpersonal racism, as connected to/enabled by institutional racism. A team member noted that we should not lump forms of systemic devaluation, exclusion, and oppression with getting bullied because of clothes and so on.

Actions that are biased, hostile, or violent toward others based on race are racist, and should be appropriately viewed and acted upon as such. Subsuming them under the concept of “bullying” clouds the dynamics of power at play. Similarly, harassment based on “perceived LGBT” status is reduced to “bullying,” while gender-based harassment is omitted entirely. Moreover, questions regarding “bullying” are presented separately (i.e., no question or response option to indicate experience of discrimination or harassment based on multiple social locations), thus making no space for understanding intersectional dynamics of students' experiences.

Third, both teams were consistent in their critique that BRFSS and SHS questions needed to be designed so that data collected can directly drive policy and practice. Members reiterated how these surveys need to focus on systemic (community, institutional, and policy) conditions rather than (solely or mostly on) individual behaviors or experiences. Furthermore, one team member said, “We don't need more detailed data about how Black folks experience even worse adverse childhood experiences—i.e., more toxic environments—we already know that. We need data that can help drive policy change.”

Consistently, project teams reiterated that for data to be actionable, we need to include information that yields accurate insights about the systems (infrastructure, neighborhood, family life, racism, transportation, etc.) in which people are making (or trying to make) the best choices they can. In suboptimal environments strained by systemic oppression and historical racism, presenting survey results without this larger context, while focusing almost exclusively on how individuals “need to change their behaviors,” can lead to further blaming and traumatizing communities.

Table 3 provides an example for the current BRFSS physical activity question and how that question could be modified or what additional context-relevant data need to be leveraged to provide more actionable findings. For instance, teams considered that individual physical activity behaviors should be contextualized using policy-relevant issues such as joint use agreements between schools and local officials, amount of greenspace, or percentage of jurisdiction zoned for public recreation. Ideally, federal, state, and local data systems should reflect a socioecological and intersectional approach,^19,24,25^ which in turn would allow us to more routinely couple individual-level health behaviors with family-, neighborhood-, community-, institution-, and policy-related contexts.

**Table 3.** What Is Needed to be Actionable? Behavioral Risk Factor Surveillance System Physical Activity Question

	Team-identified actionable data
**BRFSS—2015–2018 combined file physical activity question**	
During the past month, other than your regular job, did you participate in any physical activity or exercise such as running, calisthenics, golf, gardening, or walking for exercise?	[Understanding of what is preventing them from being physically active] Behavior data mapped in relation to policy-related physical activity contexts, such as Joint use agreements between schools and public entities Amount of greenspace % of jurisdiction zoned for public recreation use Density of free gym facilities as ratio of non-free ones Traffic/pedestrian injury rates Sidewalk existence and quality % of tax revenue invested in parks [This context then renders individual physical activity responses open to deeper examination and action, e.g., What is the relationship between joint use agreements and physical activity rates for a county/neighborhood? Is there a demographically comparable area w/similar level of agreements that has lower physical activity rates? Why?]

Fourth, team members were generally concerned with the low number of African American respondents across the different geographies in Oregon. They suggested African American community members should be able to participate in developing and administering the BRFSS and SHS.^11^ Due to small sample sizes, they questioned if the data make sense to the broader community or are representative and applicable beyond those that responded. Ultimately, they pointed out that the responsibility lays on OHA to lower barriers, and create welcoming spaces to bring people in.

Fifth, integration with other data sources is key to further contextualize BRFSS and SHS data. For instance, the Latinx team noted that Latinx respondents were the least likely to report having received influenza vaccination (BRFSS 2020). The Latinx team did not believe this was due to cultural values that were anti-immunization/vaccines, but due to social barriers around access to health insurance/health services and the potential cost of immunizations. They wanted contextual data to help them make sense of the self-reported BRFSS results.

As another example, team members questioned the accuracy of data generated by asking students if they participate in free and reduced lunch programs. They noted that students may not know or want to share, and that this information is already collected by other agencies (e.g., Oregon Department of Education—ODE). In addition, in some areas, the entire school population qualifies for free and reduced lunch, but students are still asked that question at the beginning of the academic year. The teams' suspicions are supported by comparison of SHS data with ODE data on free and reduced lunch status. In 2019, while the SHS (then called Oregon Healthy Teens) found that 57% of Latinx 8th graders and 61% of Latinx 11th graders received free or reduced priced lunches at school,^26^ ODE reported that about 75% of Latinx students enrolled in free and reduced lunch.^27^

Sixth, ensuring that surveys are written and translated in accessible ways can lead to more representative and reliable findings. Team members wanted to make sure the amount of time it takes to draft and translate documents into accessible language is recognized and compensated accordingly. Different literacy (and health literacy) levels should be considered. How do we make sure we are using health terms and questions that are understandable and culturally relevant? If we use a term such as “Latinx,” how do we contextualize that language?

Some Latinos may not understand (or agree with) it, so how can we be more inclusive? The Latinx team reviewed the translated (Spanish language) BRFSS and their translation methods. The group suggested an external advisory group should be established. Advisory members should come from various segments of the Latinx community, so they not only know the language but also the cultural context(s) in which specific words or phrases are used.

## Discussion

This project has important implications for the practice of conducting large-scale public health surveys aimed at generating information used for knowledge creation and policy and programmatic development. Robust community engagement must be a linchpin of scientific integrity and relevance for BRFSS and SHS data collection, analysis, interpretation, dissemination, and use. To move in that direction, efforts should include several actions described below, which are fundamental for applying data justice principles to survey modernization processes.

First, survey design needs to produce questions that focus on systemic aspects of individual experience, be properly contextualized, and yield actionable data. Questions about individual behavior and experience must be presented with the context to avoid shifting entire responsibility to the individual and misrepresenting people's experiences and victim blaming, and to highlight the role institutions play in creating, perpetuating, and exacerbating health inequities.^28^ If data are not presented in an actionable way, how can public health agencies be held accountable by the communities they are meant to serve?

Hence, we must ensure that BRFSS, SHS, and other survey systems ask relevant contextual questions so that their reports more accurately represent the experiences of communities of color. The fact that the types of questions asked in the BRFSS and SHS fall short of providing important contextual details is well known.^5^ Recent research shows that, while public health surveillance and monitoring systems are integrating racism as a construct, they are still primarily ask individual-level questions.^29^ Our survey modernization process demonstrates that asking more relevant questions can be done with the active involvement of communities of color, acknowledging their knowledges and lived experiences. Moreover, centering communities of color is critical toward preventing further harm.

Second, it is important to build in time and resources necessary for relationship development between governmental public health and community partners in any surveillance/survey system, including long-term, sustained, and compensated community-led data collection and reporting efforts. Integrating and sustaining community leadership throughout all aspects of the survey cycle, from design through dissemination is a step toward sharing power with communities of color, centering their expertise, and visualizing their role as architects of their own health equity efforts.^30,31^

## Conclusions

This work highlights Oregon's efforts to meaningfully incorporate community perspectives into relevant large-scale public health survey systems. Similar efforts are also slowly starting to happen in other states^31^ and at the federal level.^29^ A fundamental lesson from this work is that communities must be centered in all phases of survey modernization, from instrument design, data collection, analysis, and dissemination to decisions about how data about their communities will be used. Currently, findings from this project are being disseminated among OHA units, other agencies, and community partners, and several recommendations are being implemented (e.g., formation of a Youth Data Council—YDC).^32^

The YDC (which started meeting in March 2022) is a new advisory unit within OHA to guide the OHA Public Health Division and the ODE on how to improve the SHS across content, analysis, reporting, and communication components, ensuring that the SHS is asking questions that are meaningful and relevant to students, their families, and communities across Oregon schools. The YDC is youth led and grounded in principles of youth-adult partnership. Two successes to date include the following: (1) The 2022 SHS included open-ended questions to provide greater context and inform policy and (2) the 2023 YDC cohort developed a video, analyzed data, and held a data party with the CCC researchers guiding coding, analysis, writing, and communication of findings with an equity lens.

In summary, meaningful community leadership is needed for public health survey systems to provide more actionable pathways toward improving community health outcomes.^1,16,22^ Following a data equity approach in our survey systems would greatly move us away from a primarily individual-focused behavioral approach.^19,20^ Efforts in Oregon may help to inform similar efforts in other states and at the national level. To uphold our commitment to health equity,^23,31^ public health survey systems need to become more context oriented, actionable, and accountable to the communities they serve.

## Abbreviations Used

BRFSS: Behavioral Risk Factor Surveillance System
CCC: Coalition of Communities of Color
CCO: Coordinated Care Organization
CDC: Centers for Disease Control and Prevention
COVID-19: coronavirus disease 2019
HMOs: Health Maintenance Organizations
ODE: Oregon Department of Education
OHA: Oregon Health Authority
OHP: Oregon Health Plan
RTR: Reactions to Race
SHS: Student Health Survey
YDC: Youth Data Council
